# Home delivery and newborn care practices among urban women in western Nepal: a questionnaire survey

**DOI:** 10.1186/1471-2393-6-27

**Published:** 2006-08-23

**Authors:** Chandrashekhar T Sreeramareddy, Hari S Joshi, Binu V Sreekumaran, Sabitri Giri, Neena Chuni

**Affiliations:** 1 Department of Community Medicine, Manipal College of Medical Sciences, Pokhara, Nepal; 2 Department of Pediatrics, Manipal Teaching Hospital, Manipal College of Medical Sciences, Pokhara, Nepal; 3 Department of Obstetrics and Gynecology, Manipal Teaching Hospital Manipal College of Medical Sciences, Pokhara, Nepal

## Abstract

**Background:**

About 98% of newborn deaths occur in developing countries, where most newborns deaths occur at home. In Nepal, approximately, 90% of deliveries take place at home. Information about reasons for delivering at home and newborn care practices in urban areas of Nepal is lacking and such information will be useful for policy makers.

**Methods:**

A cross-sectional survey was carried out in the immunisation clinics of Pokhara city, western Nepal during January and February, 2006. Two trained health workers administered a semi-structured questionnaire to the mothers who had delivered at home.

**Results:**

A total of 240 mothers were interviewed. Planned home deliveries were 140 (58.3%) and 100 (41.7%) were unplanned. Only 6.2% of deliveries had a skilled birth attendant present and 38 (15.8%) mothers gave birth alone. Only 46 (16.2%) women had used a clean home delivery kit and only 92 (38.3%) birth attendants had washed their hands. The umbilical cord was cut after expulsion of placenta in 154 (64.2%) deliveries and cord was cut using a new/boiled blade in 217 (90.4%) deliveries. Mustard oil was applied to the umbilical cord in 53 (22.1%) deliveries. Birth place was heated throughout the delivery in 88 (64.2%) deliveries. Only 100 (45.8%) newborns were wrapped within 10 minutes and 233 (97.1%) were wrapped within 30 minutes. Majority (93.8%) of the newborns were given a bath soon after birth. Mustard oil massage of the newborns was a common practice (144, 60%). Sixteen (10.8%) mothers did not feed colostrum to their babies. Prelacteal feeds were given to 37(15.2%) newborns. Initiation rates of breast-feeding were 57.9% within one hour and 85.4% within 24 hours. Main reasons cited for delivering at home were 'preference' (25.7%), 'ease and convenience' (21.4%) for planned deliveries while 'precipitate labor' (51%), 'lack of transportation' (18%) and 'lack of escort' during labor (11%) were cited for the unplanned ones.

**Conclusion:**

High-risk home delivery and newborn care practices are common in urban population also. In-depth qualitative studies are needed to explore the reasons for delivering at home. Community-based interventions are required to improve the number of families engaging a skilled attendant and hygiene during delivery. The high-risk traditional newborn care practices like delayed wrapping, bathing, mustard oil massage, prelacteal feeding and discarding colostrum need to be addressed by culturally acceptable community-based health education programmes.

## Background

Of the approximately four million global neonatal deaths that occur annually, 98% occur in developing countries, where most newborns die at home while they are cared by mothers, relatives, and traditional birth attendants [1]. During the past two decades infant mortality rate has declined very slowly. This is as a result of a very slowly declining neonatal mortality rate. Despite proven cost-effective solutions to reduce neonatal mortality, such as promoting tetanus toxoid immunisation, skilled attendance during delivery, immediate and exclusive breast-feeding, and clean cord care, there has been relatively little change in neonatal mortality [2,3]. In Nepal, approximately 90% of births occur at home. In 2005, the infant mortality rate in Nepal was 64 per 1000 live births and the neonatal mortality rate was 39 per 1000 live births [4]. In rural areas of Nepal, the proportion of institutional deliveries is as low as four percent [5]. Even in urban areas like Kathmandu, a significant proportion of women (19 %) still deliver at home [6]. This is inspite of a relatively easy access to institutional maternity services in urban areas. Previous studies about home deliveries in urban and peri-urban areas of Kathmandu have reported poor maternal education, multiparity and low socioeconomic status as the predictors of home deliveries [6,7]. A study carried out in Kathmandu reported 'cost' and 'convenience' as the reasons for delivering at home [6].

The World Health Organisation's (WHO) guidelines for essential newborn care include the following: hygiene during delivery, keeping the newborn warm, early initiation of breast-feeding, exclusive breast-feeding, care of the eyes, care during illness, immunisation and care of low birth-weight newborns [8]. Therefore it is necessary for the mother and her family to understand these aspects of childbirth and newborn care and be prepared to react for the potential dangers signs. A study from rural areas of Makawanpur district, Nepal reported that a very large proportion (>90%) of deliveries took place at home. The study also reported that only six percent of home deliveries were attended by skilled government health workers and newborn care practices were unhygienic and high-risk [9]. Such high-risk practices have also been reported from remote Jumla district, Nepal [10]. Newborn care practices may change over time and may be different in urban areas. A study from low socioeconomic settlements of Karachi, Pakistan has reported that traditional newborn care practices were high-risk and emphasized the need for community-based interventions to promote proper newborn care practices in urban areas [11].

Implementation of an effective program for promotion of childbirth and newborn care practices requires understanding of the community and household traditional newborn care practices. Such information will enable the development of programs to promote culturally sensitive and acceptable change in practices. Information about the reasons for delivering at home is also necessary for healthcare planners to design appropriate maternity services. Information about reasons for delivering at home, home delivery and newborn care practices in urban areas of Nepal is lacking. Therefore, we undertook this study in an urban population of western Nepal with the following objectives:

1) to describe the home delivery and newborn care practices and

2) to assess the reasons for delivering at home.

## Methods

### Study setting

Nepal has a population of about 23 million people, 80% of whom live in rural areas [4]. With an estimated per capita income of $289 per year, Nepal is a poor developing country in south Asia [12]. Life expectancy at birth has increased, but at 60 years it is still lower than neighboring south Asian countries. Infant mortality rate is amongst the highest in the region. Due to high maternal mortality rate, life expectancy for women is lower than that for men. Gender disparities are also common in terms of literacy. Only 26% of Nepal's women are literate compared to 62% of men [13]. Curative and preventive health care are organized primarily by the Ministry of Health through hospitals located at central, regional and district levels, and primary health centres, health posts and sub-health posts located at the community level. Private hospitals and clinics exist mostly in urban areas. Missionary and not-for-profit hospitals also operate in a few areas [14]. Nepal spends about five percent of its gross domestic product on health of which only one quarter comes from the public sector and the remainder is paid for by the individual households [15].

Kaski is one of the 14 districts in the western development region of Nepal. The district has a land area of 2017 square kilometers and a population of 380,527. Kaski district has 43 village development committees and Pokhara sub-metropolitan city whose population is 156,312 [16]. Pokhara sub-metropolitan city is administratively divided into 18 municipal wards. In each of these wards immunisation clinics are conducted once a month in the child health centres. The child health centres are managed by the Urban Basic Services of Pokhara Municipal Corporation, United Nations Children's Fund (UNICEF) and Manipal College of Medical Sciences (MCOMS) each providing manpower, vaccines, medicines and technical input respectively. Since primary immunisation in Nepal is completed at one year of age, majority of the children attending these clinics were infants. A few children who missed measles vaccine between nine and 12 months are older than a year when they attend the immunisation clinic.

### Study design and participants

The institutional ethics committee of MCOMS approved this study. A cross-sectional study was carried out in the immunisation clinics of Pokhara city. We included the mothers of all the infants who were brought for immunisation during the months of January and February, 2006. The framework used for the design and presentation our study was based on a similar study carried out earlier in a rural area of Nepal [9]. A semi-structured questionnaire was developed for the purpose of this study and was pre-tested among 25 mothers during the month of January, 2006. After pre-testing, the questionnaire was modified according to local traditions and cultural sensitivity. The questionnaire sought information about sociodemographic characteristics of the family, planned or unplanned home delivery, reasons for delivering at home, the details of events that took place at home from the onset of labor pains till delivery and after birth till initiation of breast-feeding. The details included attendance at delivery, cleanliness and hygiene practices during delivery, thermal control and infant feeding. The information about the reasons for delivering at home was sought by both open and closed-ended questions. Those women who reported that they had decided to deliver in a hospital but could not reach the hospital after the onset of labor pains due various reasons were categorized as unplanned home deliveries. Those women who reported that they had decided to deliver at home were categorized as planned home deliveries.

Two health workers were trained to administer the questionnaire during a one -day training conducted by the investigators at MCOMS. The health workers were stationed at the registration counter and enquired from the mothers about place of delivery. The mothers of the infants who reported that they delivered at home were invited to participate. Verbal consent was sought and the respondents were assured that the interviewers were not a part of the health service team and services will not be denied if they declined to participate in the interview. After obtaining verbal consent, the health worker carried out the interview and recorded the necessary information on a semi-structured questionnaire. All the interviews were supervised by the chief investigator and a staff nurse who trained the interviewers. The data were coded and analyzed using the SPSS package (Statistical Package for Social Sciences) [17]. Frequencies and percentages of different variables were calculated. Chi square test was used to test if the observed differences between panned and unplanned deliveries were statistically significant. A p value of 0.05 was considered as significant.

## Results

Out of the 1320 infants who were brought to the immunisation clinics during the study period, 246 (18.6%) were born at home. Six infants were brought by a family member other than the mother or a relative. The information provided by these respondents was not reliable. Hence they were excluded from the analyses. One hundred and forty (58.3%) of these 240 home deliveries were planned whereas 100 (41.7%) were unplanned.

### Sociodemographic profile of the respondents

The median age of the infants was four months (interquartile range, 4 months). One hundred and twenty two (50.8%) infants were males and 118 (49.2%) were females. The median age of the mothers was 24 years (interquartile range, 7 years). The majority of the mothers were Hindus (196, 81.7%) followed by Buddhists (33, 13.8%). Ninety three (38.8%) respondents were illiterate and only 53 mothers (13.7%) had education of high school and above. The mean monthly family income was 6360 Nepalese rupees (Approximately 90 USD).

### Antenatal care and past obstetric performance of the respondents

Out of the 240 mothers interviewed, 73 (30.4%) had not gone for any antenatal visit and only 25 (10.4%) mothers had at least four antenatal visits as recommended by the National Safe Motherhood Program of Nepal. The majority of women received antenatal care from the publicly funded Western Regional Hospital, Pokhara. Seventy (29.2%) mothers did not receive tetanus toxoid vaccine during their previous pregnancy and 86 (35.8%) received two doses of tetanus toxoid as recommended by the National Safe Motherhood Program. Of the 173 multiparous women, 148 (85.5%) had delivered at home at least once before. Only 55 mothers (24.6%) had at least one institutional delivery in the past. Seven mothers reported of having had a still birth (2.9%), 16 a neonatal death (6.7%) and four a post-neonatal death (1.7%) after their previous home deliveries.

### Birth place and attendance at delivery

The majority (92.5%) of the deliveries took place either in a separate room or inside the house and the remaining 18 deliveries (7.5%) took place outside the house, either in the backyard or other places. One hundred and twenty eight (53.3%) deliveries were attended by neighbors, 51 (21.3%) were attended by family members and 38 women (15.8%) gave birth alone. Only 15 (6.3%) deliveries were attended by skilled personnel i.e. auxiliary nurse midwife or health assistant and 13 (5.4%) deliveries were attended by traditional birth attendants (Table 1).

**Table 1:** Place of delivery and attendance during delivery

	**Number of births (N = 240)**	**Percentage**
**Place of Delivery**		

In a room	138	57.5
Inside the house	84	35.0
Outside the house	9	3.7
Backyard of the house	6	2.5
Other	3	1.2

Attendant during delivery*		

Neighbor	128	53.3
No attendant	38	15.8
Mother	13	5.4
Mother-in-law	12	5.0
Other family member	26	10.8
Auxiliary nurse midwife	8	3.3
Health assistant	7	2.9
Traditional birth attendant	13	5.4
Others	3	1.2

* More than one attendant may have been present

### Cleanliness and hygiene practices during delivery

Ninety two mothers (38.3%) recalled that the birth attendant/s had washed their hands and 116 (48.3%) recalled that they did not do so. Thirty two mothers (13.3%) could not remember at all. Clean home delivery kits (CHDK) are currently manufactured and distributed in Nepal. Each kit contains a plastic sheet, a clean razor blade, a surface for cutting cord, soap, and a cord tie. Forty six (19.2%) mothers responded that CHDK was used and 168 (70%) had not used one during their last delivery. The umbilical cord was cut after the expulsion of placenta in 154 (64.2%) deliveries. The umbilical cord was cut with a new or boiled blade in 217 (90.4%) deliveries and in 17 (7.1%) deliveries a sickle/household knife or an old unboiled blade was used. The stump of umbilical cord was left undressed in 177 (73.8%) deliveries. But oil was applied in 53 (22.1%) deliveries. In all the instances mustard oil was used. Applications like turmeric and antiseptics were also reported by the mothers. The newborn was often wrapped in an old washed cloth (177, 73.8%) (Table 2).

**Table 2:** Cleanliness and hygiene practices during delivery

	**Number of births (N = 240)**	**Percentage**
**Instrument used for cutting umbilical cord**		

New or boiled blade	217	90.4
Sickle	7	2.9
Household knife	6	2.7
Old unboiled blade	4	1.7
Unknown	6	2.5

**Dressing applied to umbilical stump**		

Nothing	163	67.9
Oil	47	19.6
Oil and turmeric	5	2.1
Antiseptic	2	0.8
Unknown	23	9.6

**Cloth used for wrapping the baby**		

Old washed cloth	177	73.8
Old unwashed cloth	37	15.4
New unwashed cloth	16	6.7
New washed cloth	8	3.3
Unknown	2	0.8

### Maintenance of warm chain for the newborn

Since more than 90% of the deliveries took place in a room or inside the house, information about heating of the birth place was also asked. In 137 (57.1%) deliveries the birth place was heated. In 88 out of these 137 (64.2%) deliveries birth place was heated throughout the delivery and in 23 (16.8%) instances it was heated after delivery. The heating of birth place was statistically significant between planned and unplanned home deliveries. The time taken to wrap the baby was usually prolonged. Only 100 (45.8%) newborns were wrapped within ten minutes and 233 (97.1%) newborns were wrapped within 30 minutes after birth. By one hour all the newborns were wrapped. Two hundred and twenty five (93.7%) out of 240 newborns were bathed after birth. Almost all of these newborns were bathed within six hours after birth. Nearly half of them were bathed within ten minutes, 88.9% within half an hour and 96% within one hour (Table 3). The application/massage of the newborn with oil was a common practice and 144 (60%) newborns received an oil massage any time after birth. Almost all of these newborns received mustard oil massage.

**Table 3:** Practices related to maintenance of the warm chain for the newborn

	**Number of births (N = 240)**	**Percentage**
**Heating of the birth place**		

None	103	42.9
Before birth	26	10.8
After birth	23	9.6
Throughout birth	88	36.7

**Time to wrapping the baby**		

≤ 5 minutes	33	13.8
≤ 10 minutes	100	45.8
≤ 20 minutes	205	85.4
≤ 30 minutes	233	97.1
≤ 60 minutes	240	100

**Time to bathing**		

≤ 5 minutes	35	15.5
≤ 10 minutes	113	50.2
≤ 30 minutes	200	88.9
≤ 60 minutes	216	96.0
> 60 minutes	225	100

### Newborn feeding

All the newborns were breast-fed. Clarified butter (ghee), oil, honey, sugar or animal milk was sometimes given to the newborns (37/240, 15.4%) before the initiation of breast-feeding. Ghee or oil was given to 19 (7.9%) newborns. Overall, 203 mothers (84.6%) had given colostrum or breast milk to their babies as the first feed. Thirteen mothers (5.4%) had given breast milk from other lactating mothers when there was a delay in initiation of breast-feeding. Twenty six out of 240 (10.8%) mothers had discarded colostrum before initiating breast-feeding. The rates of initiation of breast-feeding were 57.9% within one hour and 85.4% within 24 hours (Table 4).

**Table 4:** Type and timing of first feed

	**Number of newborns (N = 240)**	**Percentage**
**Newborn's first feed**		

Breast milk/Colostrum	190	79.2
Ghee or Oil	19	7.9
Breast milk from other woman	13	5.4
Honey	5	2.1
Cow's or buffalo's milk	4	1.7
Glucose water	4	1.7
Plain water	3	1.3
Formula feed	2	0.8

**Time to breast-feed**		

Immediately after birth	3	1.3
≤ 15 minutes	29	12.1
≤ 30 minutes	67	27.9
≤ 60 minutes	139	57.9
≤ 24 hours	205	85.4
≤ 48 hours	230	95.8
> 48 hours	240	100

### Reasons for delivering at home

In our study, 140 out of 240 home deliveries (58.3%) were planned and in 91 (65.0%) of these planned home deliveries the reasons cited by the mothers were 'I prefer home delivery', 'home delivery is easy and convenient' and 'all my previous deliveries were at home'. In our study, 100 (41.7%) home deliveries were unplanned. The common reasons cited for unplanned home deliveries were 'precipitate labor' (51.0%), 'lack of transportation' (18.0%) and 'lack of escort' during labor (11.0%). 'Financial problems at home' and 'worries about cost of care in the hospital' (11.3%), 'distance of the hospital' (6.7%), 'fear about hospital' (2.5%) and 'family members' preference for home delivery' (2.5%) were also mentioned as the reasons for delivering at home (Table 5).

**Table 5:** Reasons for choice of planned and unplanned home deliveries

Reason given	Planned	Unplanned	Total
Preference for home delivery	36	-	36 (15)
Home delivery is easy and convenient	30	-	30 (12.5)
All my previous deliveries were at home	25	-	25(10.4)
Hospital is too far	12	4	16(6.7)
Worries about cost in the hospital	10	8	18(7.5)
Financial problems at home	7	2	9(3.7)
Family members prefer home delivery	5	1	6(2.5)
Fear of hospital	6	-	6(2.5)
Health worker lives close to house	3	-	3(1.2)
Precipitate labor*	-	51	51(21.3)
Lack of transport during labor	-	18	18(7.5)
Lack of escort during labor	-	11	11(4.6)
Onset of labor before the expected date	-	4	4(1.7)
Other reasons	6	1	7(2.9)
Total	140	100	240

Figures in parentheses indicate percentage

* Precipitate labor: Labor that results in rapid expulsion of fetus

## Discussion

The present study highlights that home deliveries are not only common in rural areas but also in urban areas where maternity services are relatively easily accessible. An earlier study from rural Nepal reported that 93% of the deliveries took place at home [9]. The proportion of home deliveries in the present study is similar to that reported from earlier studies in Kathmandu and its surrounding areas. These studies reported that the proportion of home deliveries increased, the farther one gets from urban areas [6,7].

Interestingly, some findings of the present study are similar to the previous study from rural Nepal [9]. It was surprising that skilled attendance of government health workers or traditional birth attendants, use of CHDK and hygiene practices during delivery was low in urban areas also. Practices like heating the birth place, applying mustard oil to the stump of umbilical cord, and bathing the baby soon after birth were common in urban areas. Early initiation of breast-feeding, use of prelacteal feeds and breast-feeding from another woman are also common practices prevalent in urban areas.

### Attendance during delivery

Most deliveries took place either in a separate room or some place inside the house which is similar to the report from an earlier study [9]. An earlier study has highlighted that cattle-shed deliveries were contributing to higher rates of infant mortality in the remote rural areas of Nepal [18]. Such practices were not reported in our study. Studies from rural areas have underscored the role of mother-in-law for assistance during the delivery and care of newborn [9,10]. But in our study, mother-in-law was present during delivery in only a small proportion (5%) of home deliveries. More than half of the deliveries were attended by neighbors. Such a difference may be due to demographic structure of the urban population in which many families may be economic migrants and nuclear families. Earlier studies have confirmed the extremely low presence of skilled government health staff or traditional birth attendants during delivery in rural areas of Nepal [9,10,19,20]. Maternal and child health workers who are identified as key birth attendants by the policy makers were not present at delivery in our study either. This study highlights that skilled attendance at home deliveries is very low in urban areas also. Previous studies found that about 15% of the mothers had delivered alone at home [9,19,20]. This may emphasise the low status of women in the society and the gender inequities in health. For many of these urban families pregnancy and the process of childbirth may not be a concern or priority. It takes huge efforts to change this tradition of home deliveries and lack of skilled attendance during delivery in home setting. There is an ongoing debate about reinforcing home-based birthing strategies with skilled attendants in developing countries [21]. A recent study from Nepal suggested that such a strategy might cost a substantial amount [22]. Hence there is a need for research comparing the feasibility, cost-effectiveness, acceptability, and equity implications of skilled home-based and facility-based obstetric care [21].

### Hygiene and thermal control

A study from India has reported that Infection accounts for up to 40% of neonatal deaths [24]. Therefore the WHO emphasizes on five cleans during the delivery. The 'five cleans' are: a clean place; a clean surface; clean hands; clean cord and dressing; and a clean tie. In our study only one-third of the attendants had washed their hands before delivery which is less than previous reports from rural area [9]. CHDK was used only in 19% of the deliveries which is higher than earlier reports [9,19,23]. A qualitative study from rural areas of Nepal reported that despite perceived usefulness and awareness, the use of CHDK was low, and the common reasons cited for their non-use were lack of awareness about the kit or difficulty in procuring a kit locally. The study also reported that CHDK had a limited influence on general hygiene practices [23]. In our study, despite the low usage of CHDK, new/boiled razor blade was used to cut the cord in a majority (90.4%) of deliveries. This practice is encouraging as compared to practice in rural areas where sickle or wooden knife was used in nearly one-third of deliveries and old/unboiled blade in 23% [9]. This practice was complemented by leaving umbilical stump undressed which is similar to the practice in rural areas [9]. The practice of applying unsterile substances like oil or ghee is more important risk factor than the means of cutting the cord as reported in earlier studies [25,26]. The common substances applied to the cord were mustard oil, turmeric and disinfectants. This practice was similar to the reports from earlier studies from rural Nepal and urban settlements of Karachi, Pakistan [9,11,26].

The WHO has focused on thermal control of newborn in the essential newborn care [27]. Previous studies from Nepal, India and Bangladesh have reported on health beliefs about pregnancy and childbirth. The common view is that pregnancy is a 'hot' state and postpartum is a 'cold' one [28-30]. Neonatal hypothermia in Nepal has been described earlier [31]. In our study, we found that in 46% of the instances birth place was heated during or throughout the delivery. This proportion is less than that observed in a rural study [9]. The practice of waiting for the placenta to deliver before cutting the umbilical cord was observed in 64% of the deliveries, a rate similar to that reported in a qualitative study. [28]. This practice delays immediate wrapping of the baby. This was further compounded by bathing the baby soon after birth which seems to be a universal practice. In our study, 96% of the newborns were given a bath within one hour after delivery. Similar practices have been reported earlier [9,11].

Application/massage of mustard oil to the newborn is a well established practice in Nepal [26]. In our study, 60% of the newborns received a mustard oil massage soon after birth as in an urban population of Pakistan [11]. Recently there have been increasing concerns about this traditional practice of massage with mustard oil as it is thought to predispose the newborn to the risk of hypothermia. Earlier studies have raised concerns about the traditional practices of applying mustard oil to the umbilical cord stump and mustard oil massage after delivery [26,32].

### Infant feeding

In our study the only traditional newborn care practice which seems to be healthy and encouraging is breast-feeding. As reported in the earlier studies, rates of initiation and exclusive breast-feeding are high [9,33-35]. However, practices like prelacteal feeding and discarding colostrum which still persist in urban areas are a cause of concern. Qualitative studies suggest that the traditional practice is to give a taste of non-breast milk food and usually only once [28]. In our study, 15% of the newborns received a prelacteal feed which is similar to that reported previously [9,33]. In our study, only ten percent of the mothers discarded colostrum which was in contrast to 40% in rural area [9]. Use of formula feeds was minimal and feeding with bottle and nipple almost non-existent in Nepal [33]. A recent qualitative study from rural Nepal reported that grand mothers held colostrum in high regard did not use prelacteal feeds and also supported early initiation of breast-feeding [34]. These findings have positive implications on child nutrition.

### Reasons for delivering at home

Studies carried out in Kathmandu and its surrounding areas have reported socioeconomic status and multiparity as strong predictors of the place of delivery. In our study the reasons for planned home deliveries were related to 'preference' for home delivery and perception of home deliveries as 'easy' and 'convenient' and experience of previous home deliveries. For unplanned home deliveries the reasons cited were 'precipitate labor' and 'lack of transport' and 'lack of escort' during labor. Similar findings were reported from an earlier study in Kathmandu [6]. In urban areas there is a mix of traditional families and recent economic immigrant families. In rural areas women have a strong cultural preference for home deliveries because institutional deliveries are inaccessible. This could be the reason for women indicating 'preference' as the reason for delivering at home. The decision making process in the family about the place of delivery is also an important aspect about the reasons for home deliveries. We could not explore the details of this aspect in our study. Therefore we are planning to undertake an in-depth qualitative study to explore the reasons for delivering at home.

The findings of our study suggest that easy access to maternity services may not be enough to ensure the use of such services. Lack of utilization may be influenced by income, education and cultural beliefs. In Pokhara, institutional delivery facilities are available at the Western Regional Hospital, Manipal Teaching Hospital of MCOMS and a few private hospitals. Since the services at all these facilities have to be paid for, financial constraints may be the main reason for not using these facilities. A large section of this urban population may be recent economic migrants from rural areas. This may be the reason for urban-rural similarities observed in home delivery and newborn care practices in our study.

In our study, 70% of the home deliveries were unplanned. These women would have sought institutional delivery if an ambulance service or local facility for delivery was made available. In this respect it may be worth investing on satellite maternity services run by midwives. Mothers might prefer to utilise such local and user-friendly services than a tertiary care hospital. Also the mothers need to have information about how to access a trained traditional birth attendant or a midwife during the delivery.

The National Safe Motherhood Program of Nepal emphasizes the provision of round-the-clock emergency obstetric services including transport and financial assistance. The program has recently implemented the scheme of financial incentive for the mothers who choose institutional delivery [4]. In our study, one-third of the mothers had not gone for antenatal checkups and only a third of them received two doses of tetanus toxoid during their previous pregnancy. There may be many cultural constraints for use of maternity services e.g. decision of the husband or mother-in-law which often over-rides that of the mother. The reasons for low uptake of maternity services in the urban population may be due to socioeconomic and cultural factors [6,7]. Therefore interventions should address not only the medical problems but also need to deal with wider social problems. Interventions should also focus to improve the status of women in society including increasing female literacy and empowerment to tackle the maternal health problems [36]. A recent qualitative study carried out in Kathmandu reported that attitudes of pregnant women, husbands and service providers were favorable towards encouraging greater male involvement in maternal health services [37]. Further studies are needed about quality of the available maternity services and cultural beliefs about pregnancy and childbirth.

The present study may have both selection and information bias. Since our survey was carried out in immunisation clinics, selection bias cannot be ruled out. We might have missed to interview those mothers who delivered at home and did not attend immunisation clinics. However, the sample of mothers interviewed may be representative of the urban population since the immunisation coverage is more than 90% in urban areas [4,20]. The high infant mortality rate in study area means that ten percent of the children who were born at home may have not reached their first birthday. Therefore, ten percent of the mothers did not attend the immunisation clinics. We included only those deliveries that took place within one year in order to avoid recall bias over a longer period of time. However, we cannot rule out some amount of recall and reporting bias. Hence the interpretation and generalisability our findings may be limited.

In our study, all the women who had delivered at home agreed to participate in the study. The interviewers identified themselves as independent researchers rather than as a part of health service team present in the immunisation clinics. The respondents were assured of providing health services regardless of their decision to participate in the interview and verbal consent was sought. All the women agreed to participate in the study after receiving such an assurance. The possibility that the women considered interviewers as a part of health service team cannot be ruled out. The mean monthly family income of the respondents was approximately US$90. But the annual per capita income of Nepal is US$ 289 [12]. The reported monthly family income of this urban population is higher than national average. The families in this urban population may be more affluent than an average Nepali family since Nepal is an agrarian economy. In Nepal, majority of the population is mainly dependent on subsistence farming with seasonal migration to India or other countries.

Despite the above-mentioned limitations, our study has obtained important information about home delivery and newborn care practices and reasons for delivering at home. This information has many policy implications about the ongoing safe motherhood and child survival programmes in Nepal. There need to be more focus on the skilled attendance and hygiene during delivery and the use of CHDK in urban population also. Some high-risk newborn care practices like delayed wrapping, immediate bathing, mustard oil massage, applying mustard oil to the cord; prelacteal feeding and discarding colostrum need more attention. This information will assist in planning public health interventions to change the behaviour. Expanding skilled attendance during delivery is an important issue since these urban women 'prefer' home deliveries and home deliveries are perceived as 'easy' and 'convenient'.

## Conclusion

There is a need for community-based interventions to improve the uptake of publicly-funded maternity services. Health promotion interventions are required to improve the number of families engaging a skilled attendant and hygiene during delivery. High-risk traditional newborn care practices need to be addressed by culturally acceptable community-based health education programmes to improve newborn care practices.

## Competing interests

The author(s) declare that they have no competing interests.

## Authors' contributions

CTS contributed to the design and protocol of the study, participated in the data collection, was the primary researcher and drafted the manuscript for publication.

HSJ helped with the design of the study and development of questionnaire assisted in preparation of first draft

BVS Designed and conducted the data analysis and assisted in manuscript preparation.

SG Helped in preparing questionnaire, training and supervise health workers during data collection

NC conceived the study, set up the design and criticized the earlier drafts of the manuscript

All authors read and approved the final manuscript for submission for Publication.

## Pre-publication history

The pre-publication history for this paper can be accessed here:


